# Improving Survival in Decompensated Cirrhosis

**DOI:** 10.1155/2012/318627

**Published:** 2012-07-02

**Authors:** Amar Nath Mukerji, Vishal Patel, Ashokkumar Jain

**Affiliations:** ^1^Liver Transplant Program, Department of Surgery, Temple University Hospital, 3401 N Broad Street, Suite C640 (Parkinson Pavillion), Philadelphia, PA 19140, USA; ^2^Hepatology Service, Department of Medicine, Temple University Hospital, 3401 N Broad Street, Suite C640 (Parkinson Pavillion), Philadelphia, PA 19140, USA

## Abstract

Mortality in cirrhosis is consequent of decompensation, only treatment being timely liver transplantation. Organ allocation is prioritized for the sickest patients based on Model for End Stage Liver Disease (MELD) score. In order to improve survival in patients with high MELD score it is imperative to preserve them in suitable condition till transplantation. Here we examine means to prolong life in high MELD score patients till a suitable liver is available. We specially emphasize protection of airways by avoidance of sedatives, avoidance of Bilevel Positive Airway Pressure, elective intubation in grade III or higher encephalopathy, maintaining a low threshold for intubation with lesser grades of encephalopathy when undergoing upper endoscopy or colonoscopy as pre transplant evaluation or transferring patient to a transplant center. Consider post-pyloric tube feeding in encephalopathy to maintain muscle mass and minimize risk of aspiration. In non intubated and well controlled encephalopathy, frequent physical mobility by active and passive exercises are recommended. When renal replacement therapy is needed, night-time Continuous Veno-Venous Hemodialysis may be useful in keeping the daytime free for mobility. Sparing and judicious use of steroids needs to be borne in mind in treatment of ARDS and acute hepatitis from alcohol or autoimmune process.

## 1. Introduction

Mortality in cirrhosis is usually a consequence of decompensation or its ensuing complications. Notable exceptions are cases with hepatocellular carcinoma, where death could result from local mass effects or metastatic disease. Quite often the patient has more than one feature of decompensation and presents a complex challenge. The treatment of choice for decompensated cirrhosis is orthotopic liver transplantation and many such patients are placed on transplant waiting lists. This implies that to improve mortality in this population, we need to prolong survival until a transplant is received [1]. Merion et al. [2] reported a waiting list mortality of 25% (190 out of the 760 patients in their series), which justifies the focus on this population. The MELD (Model for End Stage Liver Disease) score is used for organ allocation in the United States [3]. Although the MELD score predicts 90-day mortality based on bilirubin, INR (international normalized ratio) and serum creatinine, the predisposing factors for death and final events leading to mortality needs consideration. In this paper, we shall attempt to identify decompensated cirrhosis patient at high risk for mortality, the events leading to mortality and examine ways to prolong life until transplantation is possible.

## 2. Liver Transplantation in Perspective

Liver transplantation dramatically turns the course of disease in decompensated cirrhosis. Most patients eventually return to productive employment with minimal limitations.

However, organs are extremely scarce and there is an overwhelming mismatch in the number of potential recipients on transplant waiting lists and available donors. At the time of writing, as per data from the Organ Procurement and Transplantation Network (OPTN), there were 16,066 candidates on the liver transplant waiting list in the United States. In 2011, 6,341 liver transplants were performed: 6,094 deceased donor and 247 living donor [4, 5]. Despite attempts to increase the donor pool by increasing public awareness and successfully recruiting more marginal organs, the demand significantly outstrips the supply. Managing listed patients with increasing wait times becomes a major challenge.

## 3. MELD Score

Historically, liver allocations in the US were made based on the Child-Turcotte-Pugh (CTP) criteria. Two very severe shortcomings were observed; ascites and encephalopathy were subjective judgment calls, and owing to its poor ability to quantitatively stratify disease severity, populations with very divergent prognosis was grouped in same class (it allowed only 3 categories). To overcome these shortcomings duration spent on waiting list was used to rank within classes [6]. The transplant community realized that it failed to allocate organs based on medical priority.

**Figure 1:**
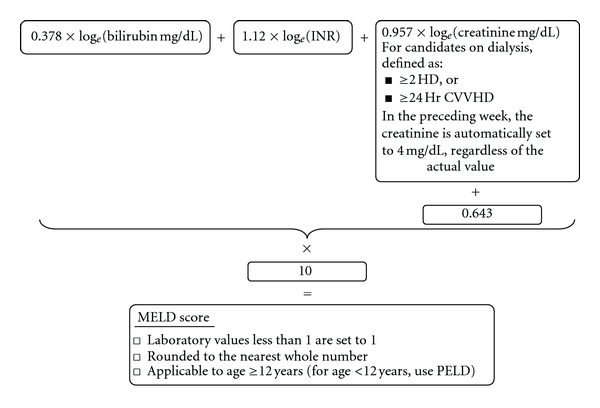
The UNOS modification of the MELD score, currently in use in the US for organ allocation.

The MELD score (see Figure 1) was originally developed at the Mayo Clinic in Rochester, to predict 90-day mortality in patients undergoing TIPS (transjugular intrahepatic portosystemic shunt) [3]. However it has proven to be useful in end-stage liver disease (ESLD) patients without TIPS as well. It is an objective and reliable indicator of the risk of short-term mortality in patients with ESLD of various etiologies and severity. It is a broad indicator of physiologic reserve in decompensated cirrhosis. Its predictive accuracy supersedes the clinical pattern of decompensation. Presence of encephalopathy, variceal hemorrhage, ascites, spontaneous bacterial peritonitis (SBP), or muscle wasting is not included in the scoring system since it failed to augment its accuracy in predicting survival, though they carry significant morbidity. Patients with MELD scores around 30 and above need particular attention. They are not only the most likely to be transplanted, but also the most likely to die if not transplanted in a timely fashion, thus presenting a very narrow window of opportunity to save them.

There is an ongoing substantial effort in the transplant community worldwide to augment and refine the MELD score. The MELD-Na, a modification including sodium levels into the score, has received much attention. Fisher et al. [7] report in their prospective trial that including exceptional points for persistent serum sodium <130 mEq/L improved organ allocation to hyponatremic cirrhotics, a high risk subset. Several authors [8–11] report that inclusion of sodium to MELD improves its predictive accuracy. However, the main criticism has been the large daily variations which can be the result of and therefore manipulated by various therapeutic measures (IV fluids, diuretics). This lack of stability has prevented the widespread adoption of sodium in MELD.

## 4. Decompensation: Definition and Mortality

In cirrhosis, the presence of any one or more of jaundice, ascites, portal hypertensive gastrointestinal bleeding, and/or, encephalopathy is considered decompensation. Gifted with an amply generous functional and regenerative reserve, the transition from well-compensated cirrhosis to symptomatic decompensation is a clinical and biochemical continuum. The above manifestations appear when the disease process overwhelms the compensatory mechanisms, either by disease progression or a superimposed acute insult. Various studies quote the rate of decompensation at 3–5% per year [12].

### 4.1. Risk of Mortality in Decompensation

Decompensation, per se, is a significant risk for mortality. One-year mortality in compensated cirrhosis is 1–3.4%, but in decompensation it is 20–57% [13–15]. Alcohol and hepatitis C account for the bulk of cirrhosis cases. Two prospective cohort studies following the natural history of alcoholic [16] and hepatitis C [17] cirrhosis have been reported on the mortality following each type of decompensation (Table 1).

Most studies concur that ascites is most frequently the first manifestation of decompensation. Although the least common (2.4–5%), encephalopathy has the highest mortality (50–65% at 5 years) [16, 17]. Therefore the management of encephalopathic decompensated cirrhosis needs special care, as it represents the subset of patients at the greatest risk of death.

In the following sections we review the management strategies for decompensated cirrhosis which may have value in improving survival.

## 5. Jaundice

Serum bilirubin is included in both Child-Pugh and MELD scores. It is readily apparent to patients and those around them. However, jaundice by itself does not lead to death.

## 6. Ascites

Uncomplicated ascites causes morbidity [18] mechanically from distension and consequent respiratory embarrassment and by increasing the risk of infection and herniae. In the extreme form, refractory ascites is associated with 1-year mortality of 28–79% [19]. There is an escalation of mortality from ascites in sepsis from SBP, hyponatremia, renal failure, and at times from obstruction/strangulation of associated herniae [20, 21]. There are two notable observations; firstly the Child-Pugh and MELD scores do not incorporate all possible predictors (MELD only includes creatinine). Secondly, these complications may be precipitated by the therapy of ascites itself (diuretics and large volume paracentesis, LVP). This calls for maintaining a fine balance between individualized therapeutic goals and alertness for early detection and aggressive correction of these complications. 

### 6.1. General Management of Uncomplicated Ascites [22–24]

**Figure 2:**
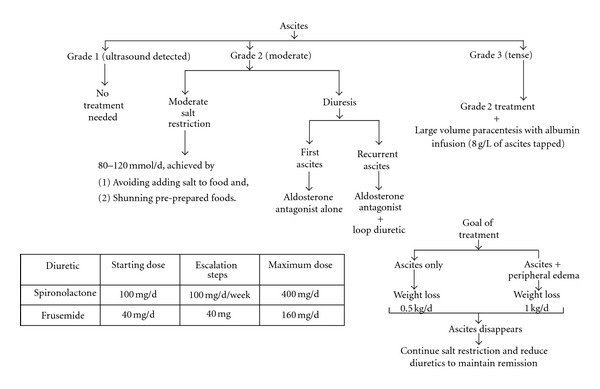
Approach to management of uncomplicated ascites.

Various measures have been suggested in managing ascites. We have compiled the plan of care after reviewing available recommendations (Figure 2).

### 6.2. Avoidance of Hypotension in Large Volume Paracentesis (LVP)

LVP can potentiate a constellation of circulatory disturbances which may result in rapid reaccumulation of ascites, dilutional hyponatremia, hepatorenal syndrome (HRS), and increased portal venous pressures. All of these can decrease survival [24]. Albumin infusion is the most effective preventable measure of postparacentesis circulatory disturbance [25]. The current consensus is to replenish 8–10 g of albumin for every liter tapped, if over 5 liters are removed (i.e., for 6 liters removed, 48–60 g of albumin is to be infused) [22].

### 6.3. Refractory Ascites

Refractory ascites was noted in 17% patients by Planas et al. [26]. The International Ascites Club defines refractory ascites as “ascites that cannot be mobilized or the early recurrence of which (i.e., after LVP) cannot be satisfactorily prevented by medical therapy” [27, 28]. Clinically, two varieties are appreciated, *diuretic resistant* (ascites unresponsive to maximal diuretic therapy) and *diuretic intractable*  (limitation of diuretic use due to development of hepatic encephalopathy, renal dysfunction, and/or electrolyte abnormality).

Patients with refractory ascites have median survival of 6 months [28]. The mainstay of treatment is repeated LVP and in selected cases, TIPS.

Deltenre et al. [29], Albillos et al. [30], D'Amico et al. [31] and Saab et al. [32], examines the role of TIPS versus paracentesis for refractory ascites by meta-analyses. They concluded that TIPS was more effective than paracentesis in clearing ascites but with equivocal effect on survival and increased incidence of encephalopathy. Rössle et al. [33] in their critical update concluded that TIPS improved survival in selected patients with refractory ascites requiring frequent LVP.

Compared to LVP, TIPS is slower to resolve ascites, but may offer a longer term solution. It is not without complications. TIPS occlude or partially thrombose in 10–80% cases. However, there is a small risk for infection. Despite improvement in refractory ascites and portal hypertension-related gastrointestinal bleeding, the diversion of blood flow can cause further hepatic decompensation and hepatic encephalopathy. Therefore, judicious discretion needs to be employed in considering TIPS in refractory ascites, especially without gastrointestinal bleeding. There should be no sign of encephalopathy, patient should be young and liver function fairly well preserved in other domains. TIPS is not recommended in advanced liver disease or in presence of prominent extrahepatic comorbidities. Usefulness of TIPS in recurrent HCV has been studied in postliver transplant patients [34].

A number of recent investigators have suggested that *β*-blockers potentially may worsen the post-paracentesis circulatory changes in refractory ascites and impoverish survival [35–40]. This may help us to refine the indication of *β*-blockers in this subset of patients who have high mortality and improve survival. It is clear, however, that further investigation is warranted.

### 6.4. Spontaneous Bacterial Peritonitis: Definition

SBP can present with local signs of peritonitis, manifestations of systemic inflammatory response, worsening of liver function, or may precipitate encephalopathy, renal failure, or gastrointestinal bleeding [41]. It may also be asymptomatic especially in the outpatient setting [42, 43]. Given this diverse clinical picture it is recommended that a diagnostic paracentesis be performed for all hospitalized cirrhotic patients with ascites and in any of the above scenarios. Diagnosis of SBP is established by ascitic fluid neutrophil count >250/mm^3^ by microscopy [24, 41]. Ascitic fluid and blood cultures should be performed, however, ascites culture is positive only 40% of the time. A negative culture does not contradict the diagnosis and should not prevent or delay treatment.

### 6.5. Spontaneous Bacterial Peritonitis: Prophylaxis

Despite optimal management of SBP, mortality is around 10–20% [44]. Transmural bacterial translocation is believed to be a predominant factor in the development of SBP, therefore prophylaxis is targeted at gut flora [45].

Acute gastrointestinal bleeding and SBP are mutually causative. Infections occur in patients with gastrointestinal haemorrhage [46–53] and presence of infection escalates rebleeding and mortality [54–58]. Antibiotic prophylaxis has been of proven benefit in this clinical scenario [55].

Patients with low ascitic fluid total protein (<1–1.5 g/dL) are at a heightened risk of SBP. Numerous studies [59–63] addressing this concur that SBP incidence is reduced by antibiotic prophylaxis, although they differ in inclusion criteria with reference to severity of liver disease. Fernández et al. [62] established a survival benefit at 3 months with antibiotic prophylaxis.

Following a first episode of SBP, cumulative recurrence rate and survival in the first year is 70% and <50%, respectively [45]. Gines et al. [64] demonstrated a reduction of recurrence of SBP from 68% to 20% with norfloxacin prophylaxis, presently the drug of choice. Other studies [59, 65, 66] have assessed alternatives including oral ciprofloxacin and co-trimoxazole. The duration of prophylaxis remains uncertain; until improvement in liver function or through to transplantation.

Frequent organisms isolated include Gram-negative bacteria (GNB), usually *Escherichia coli* and Gram-positive cocci (GPC), mainly streptococcus species and enterococci [41, 67, 68]. Fernández et al. [68] report that 30% of isolated GNB are resistant to quinolones and 30% are resistant to trimethoprim-sulfamethoxazole; 70% of quinolone-resistant GNB are also resistant to trimethoprim-sulfamethoxazole. Quinolone-resistant GNB is more frequent in patients on norfloxacin prophylaxis. Fortunately, cephalosporin-resistant isolates are unusual despite of norfloxacin prophylaxis. GNB predominate in community-acquired case, whereas GPC prevails in the hospital acquired population.

### 6.6. Spontaneous Bacterial Peritonitis: Management

Empirical antibiotics are recommended at clinical suspicion or after a raised cell count following paracentesis without waiting for cultures to result [24, 41]. The agent of choice is cefotaxim, a third generation cephalosporin, with co-amoxiclav and quinolones being alternatives [22, 24, 69]. Resolution should be documented by periodic repeated paracentesis, until ascitic fluid neutrophil count is <200/mm^3^ and cultures are sterile [24]. Failure of treatment is seen in less than 10% of patients and is managed by ruling out secondary peritonitis and changing antibiotics per culture and sensitivity results if available or to an alternative broad spectrum empiric option [22].

### 6.7. Hyponatremia

Hyponatremia is associated with high mortality in decompensated cirrhosis [70, 71]. When included, serum sodium amplifies the predictive accuracy of MELD in foretelling mortality in liver failure [9, 72]. By itself, hyponatremia is an independent predictor of fatality in decompensated cirrhosis [73]. Pathophysiologically and therapeutically hyponatremia in cirrhosis can be hypovolemic or hypervolemic. The former results from overuse of diuretics and can usually be treated by discontinuing the drug and fluid support. The latter is in a large part due to nonosmotic ADH-induced free water retention from the hemodynamic disturbances in cirrhosis. The mainstay of treatment is diuretic discontinuation, free water restriction, vaptans, and albumin.

The general agreement amongst experts is to treat hyponatremia in cirrhotics when serum sodium falls below 130 mEq/L, thus making a concession for the long standing, slowly developing, and usually asymptomatic low baseline sodium in this population.

Free water restriction to 1–1.5 L/day prevents further fall in sodium level but alone is usually insufficient to correct low levels [74]. Although albumin has been shown to improve serum sodium levels, it may not be a viable long-term solution [75]. Vaptans are distal tubular V2 receptor antagonists. Recently, Gerbes et al. [74] and Wong et al. [76] with lixivaptan, Schrier et al. [77] with tolvaptan, and Ginès et al. [78] with satavaptan demonstrated clinically and statistically significant amelioration of low serum sodium levels in a large majority of the sample studied despite heterogeneity in duration of treatment and study design.

### 6.8. Hepatorenal Syndrome

HRS refers to renal failure in advanced cirrhosis in the absence of any identifiable cause. It is therefore a diagnosis of exclusion. Common causes of renal failure in liver disease include hypovolemia, shock, use of nephrotoxic drugs or concomitant intrinsic renal disease. The International Ascites Club delineates HRS into a Type 1 syndrome, which is rapidly progressive (doubling of baseline serum creatinine to >2.5 mg/dL in <2 weeks) and Type 2, which is more indolent [28].

HRS is associated with a very dismal prognosis. Overall survival is around 3 months [79] and left untreated median survival may be as low as 1 month [80]. Type II HRS has a somewhat better prognosis than Type I, with median survival being around 6 months [81]. Subjects with HRS have a higher mortality while waiting for transplantation, which is the only definitive treatment.

Type I HRS is commonly triggered by a bacterial infection, usually SBP [82–85]. Type II HRS is commonly seen in the setting of refractory ascites. Around 30% patients with SBP develop HRS [82]. Antibiotics coupled with albumin in treating SBP has been found to diminish chances of HRS and improve survival [82]. The avoidance of hypotension following LVP decreases chances of precipitating HRS as well.

Although transplantation is the definitive treatment [22], other options serve to support the wait before the transplant. Although chronic hypotension and consequent renal vasoconstriction [81, 86] contributes to the pathogenesis of HRS, the condition is volume unresponsive.

Randomized trials [87–90] document that terlipressin improves mortality in HRS, though it works about half the time. The recommended dose is 1 mg/2–6 h, escalating to 2 mg/2–6 h following a failure of reduction in serum creatinine by 30% compared to the baseline after 3 days of use. Terlipressin is continued until creatinine falls below 1.5 mg/dL measured on two consecutive days. A gradual improvement in arterial pressure, urine volume, and serum sodium concentration is noted. Median time to response is 2 weeks and is governed by the pre-treatment serum creatinine, with quicker response in lower baseline values.

The literature is divided over the use of midodrine (2.5–7.5 mg q8h), octreotide (100–200 *μ*g subcutaneously q8h) and albumin (1 g/kg on day 1 then 40 g/day) [91, 92]. Although they improve renal parameters, their effect on survival has not been sufficiently documented [93]. There is a paucity of studies appraising the role of renal replacement therapy in mitigating mortality in HRS. However, it is valuable in combating acute indications including severe acidosis, hyperkalemia or severe volume overload. The avoidance of nephrotoxic drugs and radiologic contrasts cannot be overemphasized.

## 7. Gastrointestinal Bleeding

The presence of varices parallels disease severity [22]. Chances of a gastrointestinal bleed are 12–15% per year. The mortality from each episode may be 15–20% [22]. Prophylaxis of variceal hemorrhage therefore has an important role. A substantial decline in mortality from variceal hemorrhage has been noted in the last two decades with progresses in repeated and sequential endoscopic banding, and/or sclerotherapy and critical care management.

### 7.1. Noninvasive Screening of Esophageal Varices

Endoscopy is the gold standard in screening for varices. Noninvasive methods of predicting varices are attractive as it they would avoid a significant number of endoscopies where no varices are found and enhance compliance to variceal screening programs. Moreover it would greatly reduce the cost of care and avoid the small but definite percentage of procedure related complications.

De Franchis [94] in a review examines several options, categorizing them as biochemical and ultrasound (platelet count/spleen diameter ratio, platelet count + Child-Pugh class, Fibrotest, etc.), transient elastography (FibroScan), CT scanning for varices, and video capsule endoscopy. He concludes that although not equivalent to endoscopy, platelet count/spleen diameter ratio, CT scanning, and video capsule endoscopy by possibly being more cost effective and acceptable to patients may increase compliance and potentially have an overall superior performance. Kim et al. [95, 96] report P2/MS (platelet-count)^2^/(monocyte fraction (%) × segmented neutrophil fraction (%)) and another index based on ultrasonologic liver stiffness measurement, spleen diameter and platelet count to be promising for screening. Nguyen-Khac et al. [97] conclude that the etiology of cirrhosis has a profound impact on cutoffs for FibroScan (transient elastography, a measure of liver stiffness). Other authors report thrombocytopenia, large spleen size, portal vein size, and platelet spleen diameter ratio to be strongly predictive [98, 99].

Thabut et al. [100] in their review states that most of the noninvasive markers performed well in severe cases, but were of limited utility in moderate portal hypertension. More studies are required to clarify the status of the various non-invasive modalities vis-à-vis endoscopy for screening esophageal varices.

### 7.2. Prophylaxis

Randomized trials have established that primary prophylaxis of varices does indeed decrease mortality [101]. It is recommended that following a diagnosis of cirrhosis a screening upper endoscopy be done periodically [102].

Factors identifying patients at a high risk of variceal bleeding are large variceal size, red wale marks (longitudinal dilated venules similar to whip marks on the variceal surface), and advanced liver disease [22] helping to focus prophylactic efforts.

If no varices are identified on initial screening endoscopy, it should be repeated after 3 years, earlier if the patient decompensates. If medium-to-large varices are identified, then prophylaxis is offered in the form of nonselective *β*-blockers and/or endoscopic variceal ligation. In small varices with Child class A and no high risk “red wale” sign, prophylaxis with nonselective *β*-blocker is optional. Surveillance endoscopies are recommended if the prophylaxis is not employed. In small varices with “red wale” sign or with Child class B or C, pharmacological prophylaxis is the norm [22].

A meta-analysis [103] of 16 trials with over 1,000 patients indicates that *β*-blockers and endoscopic variceal ligation may be equivalent in survival benefit. *β*-blockers may have a wider spectrum of applicability in non-variceal bleeding, including portal congestive gastropathy where the utility of therapeutic endoscopy is limited [104]. In practice both are commonly employed in the absence of contraindications.

### 7.3. Management

#### 7.3.1. Resuscitation and Supportive Therapy

Airway protection (see Section 10), transfusions for volume support, and reversal of coagulopathy and antibiotic prophylaxis form the foundation in resuscitation of acute gastrointestinal bleeding. Coagulopathy, if present, must be supported by appropriate blood product transfusion. The evidence for factor VII transfusions is equivocal [105, 106]. Either norfloxacin or ceftriaxone may be used for prophylaxis of SBP, with the latter being more effective in higher stage of liver disease [41, 107].

#### 7.3.2. Specific Treatment

Pharmacotherapy and endoscopy are the cornerstones of treatment. Trials do not demonstrate any significant difference between the available pharmacological options, namely, somatostatin and vasopressin and their analogs octreotide and terlipressin, respectively [108]. They may differ in side effect profiles. Vasopressin has been reported to cause myocardial ischaemia, infarction and even cerebrovascular accidents [108]. Variceal ligation is superior to sclerotherapy [109, 110] in hemorrhage control and prevention of rebleeding, however, survival is similar. In the event of a failure to control hemorrhage, TIPS or portosystemic shunt surgery need to be considered. TIPS, an interventional radiological procedure, is safer and is preferred [111, 112]. Surgery is reserved for uncontrolled bleeding in carefully considered Child-Pugh Class A patients with portal vein thrombosis, where TIPS will be ineffective. Balloon tamponade may be employed temporarily in failure to control hemorrhage until the patient is stable enough for TIPS or surgery. However the use of balloon tamponade may cause aspiration or perforation in as many as 20% cases, which restricts it to short-term use only.

#### 7.3.3. Hepatic Vein Portal Gradient (HVPG)

The HVPG is being studied as a very strong prognostic indicator with potential in guiding therapy. D'Amico et al. [113] found that a reduction of HVPG <12 mm Hg or a >20% decrease from baseline significantly reduced the risk of bleeding and even mortality. Ripoll [114] in their extensive review found that HVPG could potentially guide prophylaxis in varices and predict the development of various complications of portal hypertension. It was also suggested that as a representative of a larger area of the liver, HVPG could circumvent the sampling error of a liver biopsy in assessing severity of disease. Recent studies have in particular, strongly supported the role of HVPG in guiding prophylaxis and management of portal hypertensive bleed [115–117]. However, presently, the invasive nature of the procedure and logistic issues restricts availability to referral facilities and research centers.

## 8. Hepatic Encephalopathy

Hepatic encephalopathy is the most debilitating of all manifestations of decompensation but may be controllable. Usually a precipitating event is identifiable, commonly a gastrointestinal bleed, infection, dyselectrolytemias such as hyponatremia or hypokalemia, and use of sedatives, especially benzodiazepines amongst others. An aggressive search for a precipitant forms the first line of management.

A low protein diet is no longer recommended [118, 119]. Lactulose and gut flora reduction is the mainstay of management. Lactulose is usually administered orally, however, enemas may be considered in altered sensorium as colonic flora plays the greater role in pathogenesis. Oral neomycin, metronidazole and rifaximin have been examined in studies. Neomycin can have severe systemic effects, including ototoxicity and nephrotoxicity disqualifying its long term use. Metronidazole is limited by peripheral neuropathy with prolonged usage. Rifaximin is efficacious and has no significant side effects. We have used it with lactulose in severe cases when rifaximin alone has been inadequate to control encephalopathy, although it is yet to be reported.

Besides known cases of sedative use, flumazenil may be useful if the precipitant is elusive. TIPS occlusion may be attempted if there is a recurrent or refractory hepatic encephalopathy following its placement [120].

## 9. Heparin in Advanced Cirrhosis

Portal vein thrombosis (PVT) in cirrhosis is reported to occur in 10–20% of cirrhosis. The obstacle to portal inflow is thought to be an important factor [121]. There may also be a reduction in the synthesis of anticoagulant proteins early in cirrhosis, leading to a transient hypercoagulable state which may predispose to PVT [122]. Hepatocellular carcinoma must be aggressively ruled out in PVT, since as much as 34.8% incidence of portal vein thrombosis was reported in these cases [123]. PVT commonly manifests with variceal bleeding, refractory ascites, or even intestinal infarction (when the superior mesenteric vein is involved) suggesting that PVT may decrease survival in cirrhosis though data is lacking [121, 124, 125]. It is also unknown if patients with asymptomatic PVT have different survival compared to cirrhotics without PVT [121]. Once considered a contraindication, liver transplantation in portal vein thrombosis is now feasible, though with greater morbidity and mortality [126–128].

In a recent international symposium, Villa et al. [129], presented the results of their randomized trial in which they found enoxaparin safe and effective in preventing portal vein thrombosis in advanced cirrhosis. Interestingly it was noted to remarkably reduce the incidence of decompensation. The proposal merits further investigation as the results have a potential role in improving survival and also decreases the proportion of patients presenting with a difficult surgical challenge, portal vein thrombosis.

## 10. Discussion

### 10.1. Airway Protection

Aspiration is common in encephalopathy as a consequence of feeble airway reflexes. This is compounded by the impoverished nutritional status and scanty muscle mass implying borderline physiological reserve.

The mainstay of airway protection is intubation. The focus should be on avoiding situations challenging the airways and preemptive intubation in anticipation of perilous situations.

### 10.2. Low Threshold for Intubation

Patients with a high MELD score with severe hepatic encephalopathy (most of grade III and all of grade IV) should be electively intubated, especially, if a transplant is imminent.

During resuscitation we strongly recommend endotracheal intubation for airway protection especially in compromised mental status and before endoscopic attempts. The literature is replete with studies demonstrating aspiration during upper gastrointestinal bleed and endoscopy [130–132]. Such an instance would potentially lead to an absolute contraindication for transplantation and result in death. We feel it is one of the major factors precipitating death which is not well reported and is preventable. Rudolph et al. [133] specifically studied the effect of liberal intubation during endoscopy for upper GI bleed. They found that although it did not significantly decrease the incidence of pneumonia, but likely prevented the few lethal massive aspirations.

### 10.3. Preemptive Intubation: Preprocedure and for Transportation

In an encephalopathic patient already predisposed to aspiration, we recommend preemptive intubation for all routine gastrointestinal endoscopies, namely upper endoscopy, endoscopic retrograde cholangio-pancreatography (ERCP), and colonoscopies. The position of the patient, sedation, the compromised lower esophageal sphincter competence by passage of the endoscope, and the gastric and/or abdominal distension by the insufflations synergizes with the weak airway reflexes in encephalopathy resulting in prohibitive risks of aspiration and mortality. The same may be considered for other procedures needing sedation, specifically in presence of encephalopathy and particularly with MELD score >30.

It may be prudent to consider electively intubating a sick decompensated cirrhosis patient prior to transportation from a smaller facility to a liver transplant program. Decisions are based on mental status, hemodynamic status, general condition, and travel distance. Acute events during transport are potentially more fatal than while in-house.

### 10.4. Conservative Extubation

We recommend a more conservative approach in extubating patients recovering from encephalopathy after an acute need for intubation. Extubation should be attempted when the patient is fully alert and awake for a substantial duration of time. Such patients often go through repeated cycles of intubation-early extubation and reintubation. In such instances, even a small volume of aspiration can trigger ARDS and the patient may lose the window for safely undergoing transplantation.

### 10.5. Avoiding Bilevel Positive Airway Pressure (BIPAP)

BIPAP by its positive pressure mechanism increases the volume of air reaching the stomach. The presence of altered gastrointestinal motor function in cirrhosis and encephalopathy leads to stasis and sizeable residual volumes. Large ascites, if present, adds to the increased intra-abdominal pressure. Use of BIPAP in our experience causes gastric distension which along with the preexisting impaired gastric motility increases the risk of aspiration. In our practice we strongly condemn BIPAP and prefer intubation.

## 11. Cachexia

A study looking at 114, 703 inpatient admissions in the US with cirrhosis and portal hypertension documented a greater prevalence of protein calorie malnutrition (*P* < 0.0001) than in general medical inpatients. In cirrhotics with malnutrition, inhospital mortality was roughly double than in cirrhotics without malnutrition after due statistical adjustments (*P* < 0.0001) [134].

### 11.1. Muscle Wasting

Cirrhosis is characterized by elevated resting energy expenditure [135]. The pro-inflammatory cytokine milieu [136, 137], recurrent bacterial infections and endotoxemia [138] and sympathetic hyperactivity [139] have been suggested as mechanisms underlying this hypermetabolic state. Along with diminished nutritional intake and lack of physical activity it results in muscle wasting, which in advanced cases can be severe. However, the influence of protein loss from regular LVP and malabsorption from lack of bile in gastrointestinal tract with use of lactulose remains underemphasized.

### 11.2. Physical Mobilization

Physical mobilization is of cardinal importance in maintaining physiologic reserve and protection against, most notably, pulmonary infections. When faced with a life-threatening event or decompensation, a better-preserved patient has greater likelihood of survival. Although the cachexia of cirrhosis is multifactorial, the importance of mobilization cannot be overemphasized.

Regular and rigorous physical therapy plays a pivotal role in the mobilization of these patients who are otherwise resigned to their bed. In addition, we practice sending a member from our service to visit the patient periodically throughout the day to make him/her perform simple range of motion exercises for the major joints and incentive spirometry and also engage members of the family when available. This keeps the patient active throughout the day even beyond the stipulated time for physical therapy. It also generates an opportunity for communication between the patient and the caring team.

### 11.3. Nutrition: Postpyloric Feeding and Total Parenteral Nutrition TPN

Nutritional support goes hand in hand with mobilization in combating cachexia and preservation of vitality until transplantation. In our practice, the major hindrances to nutrition are:

- poor or inadequate oral intake,
- long periods off the floor/unit for various procedures,
- lactulose induced diarrhea, and
- extensive protein loss from recurrent large volume paracentesis.

If the oral/enteral intake is judged to be insufficient for the demand, we recommend a low threshold for supplementary nighttime postpyloric tube feeds and/or parenteral nutrition.

Multiple (4–6) daily servings are recommended with nocturnal meal supplementation. This has been shown to increase lean body mass over a period of time [140]. In liver failure there is early onset gluconeogenesis (consuming muscle protein and fat) following short periods of starvation during sleep, or long intervals between meals, normally is seen after 2-3 days [141].

There is an increase in gastrointestinal transit time in cirrhosis, which is multifactorial [142, 143]. This causes increased gastric accommodation and slow emptying. For critically ill patients, such as a ventilated decompensated cirrhotic with impaired gastric motility, postpyloric feeding reduces the risk of aspiration.

Especially for the encephalopathic or intubated patient, if postpyloric feeding is not possible for any reason, we avoid feeding in the stomach and prefer TPN until postpyloric tube is placed. In most instances postpyloric tubes can be successfully placed under fluoroscopic guidance [144]. Electromagnet-assisted devices are now available and are being evaluated in some centers [145, 146].

## 12. Renal Replacement Therapy at Nighttime

Cirrhosis patients with hepatorenal syndrome often need dialysis. We prefer night-time for the renal replacement therapy, so that the daytime is spared for physical activity in the subset of patients without or with well-tolerated encephalopathy and who are not on a ventilator.

Naka et al. [147] in 2004 found very encouraging results using continuous venovenous hemodialysis as the only renal replacement modality, though they studied both pre- and post-transplant patients. More studies are needed to explore this further.

## 13. Sparing and Judicious Use of Steroids

The indications of steroids in decompensated cirrhosis with superimposed acute alcoholic hepatitis need refinement [148]. The use of systemic steroids for respiratory indications should be carefully considered. We feel that steroids should be used frugally and reserved only for specific situations, as an infection in decompensated cirrhosis will preclude transplantation. We have observed early onset of Cytomegalovirus viremia following transplantation in prolonged pretransplant steroid usage.

## 14. Take Home Message: The Distillate

The 90 days predicted mortality in decompensated cirrhosis with MELD score ≥30 is 90% [3]. With every point of rise in the score the mortality increases exponentially. There is a crucial window of time when a decompensated cirrhosis patient is critically ill with a high enough MELD score to receive organ allocation, but is still a reasonable surgical risk. The key to improving survival is to preserve the patient as a suitable transplant candidate during this tight window of opportunity. Besides the established practices, we specifically emphasize the protection of airways by avoidance of sedatives, avoidance of BIPAP, elective intubation in grade III, or higher encephalopathy, maintaining a low threshold for intubation with even lesser grades of encephalopathy when undergoing gastrointestinal endoscopy or intercenter transportation. Postpyloric tube feeding should be considered in altered mental status minimizing the risk of aspiration. In nonintubated and well-controlled encephalopathy, continued physical mobility by active and passive exercises beyond the stipulated time of physical therapy, nighttime CVVHD as preferred mode of renal replacement therapy keeping the daytime free for mobility should be considered. Sparing and judicious use of steroids needs to be borne in mind in treatment of ARDS and acute hepatitis from alcohol or autoimmune process. Our recommendations are summarized in Table 2.

## Figures and Tables

**Table 1:** Presentation of decompensation and mortality.

Study	Etiology	Ascites^∗^		GI Bleed^∗^		Encephalopathy^∗^	
		Incidence	5-year survival	Incidence	5-year survival	Incidence	5-year survival
Alvarez et al. [16] (*n* = 165)	Alcohol	50.9%	47%	24.2%	61%	2.4%	50%
Planas et al. [17] (*n* = 200)	Hep C ± Alcohol	48%	40.6%	32.5%	69.6%	5%	35%

^∗^Incidence as first decompensation, as % of cohort, (5-year survival).

**Table 2:** High risk patient (high MELD Score > 30) and recommendations.

Condition	Scenario	Special recommendations
Airway protection in encephalopathy	General	(1) Avoidance of BIPAP.
		(2) Elective intubation for grade III and grade IV encephalopathy.
		(3) Low threshold for intubation.
		(4) Continue intubation if transplant likely soon.
		(5) Extubate only when convincingly awake for considerable duration.
		(6) Postpyloric feeding if intubated.
		(7) Avoidance/minimal use of sedatives and analgesics.
	Procedures (endoscopy, etc.)	Preprocedural preemptive intubation.
	Transportation	Low threshold for elective intubation for transportation to different centre.

Hepatorenal syndrome	Prolonged periods of physical inactivity form dialysis	Nighttime continuous venovenous hemodialysis keeping daytime free for mobilization

Cachexia		(1) No protein restriction.
		(2) Frequent small meals.
		(3) Nighttime meal supplement (postpyloric tube feeds or TPN if diarrhea from lactulose) to avoid triggering of muscle consuming gluconeogenesis.
		(4) Aggressive physical therapy.
		(5) Periodic visit by medical team member making patient perform simple range of motion exercises for all major joints throughout the day.
		(6) Frequent incentive spirometry in daytime.

Infections/sepsis	Ascites ± SBP, GI bleeding	(1) Aggressive screening, prophylaxis and treatment for infections.
		(2) Sparing and judicious use of steroids.
